# Biomedical informatics and translational medicine

**DOI:** 10.1186/1479-5876-8-22

**Published:** 2010-02-26

**Authors:** Indra Neil Sarkar

**Affiliations:** 1 Center for Clinical and Translational Science, Department of Microbiology and Molecular Genetics, & Department of Computer Science, University of Vermont, College of Medicine, 89 Beaumont Ave, Given Courtyard N309, Burlington, VT 05405 USA

## Abstract

Biomedical informatics involves a core set of methodologies that can provide a foundation for crossing the "translational barriers" associated with translational medicine. To this end, the fundamental aspects of biomedical informatics (e.g., bioinformatics, imaging informatics, clinical informatics, and public health informatics) may be essential in helping improve the ability to bring basic research findings to the bedside, evaluate the efficacy of interventions across communities, and enable the assessment of the eventual impact of translational medicine innovations on health policies. Here, a brief description is provided for a selection of key biomedical informatics topics (Decision Support, Natural Language Processing, Standards, Information Retrieval, and Electronic Health Records) and their relevance to translational medicine. Based on contributions and advancements in each of these topic areas, the article proposes that biomedical informatics practitioners ("biomedical informaticians") can be essential members of translational medicine teams.

## Introduction

Biomedical informatics, by definition[1-8], incorporates a core set of methodologies that are applicable for managing data, information, and knowledge across the translational medicine continuum, from bench biology to clinical care and research to public health. To this end, biomedical informatics encompasses a wide range of domain specific methodologies. In the present discourse, the specific aspects of biomedical informatics that are of direct relevance to translational medicine are: (1) bioinformatics; (2) imaging informatics; (3) clinical informatics; and, (4) public health informatics. These support the transfer and integration of knowledge across the major realms of translational medicine, from molecules to populations. A partnership between biomedical informatics and translational medicine promises the betterment of patient care[9,10] through development of new and better understood interventions used effectively in clinics as well as development of more informed policies and clinical guidelines.

**Figure 1:**
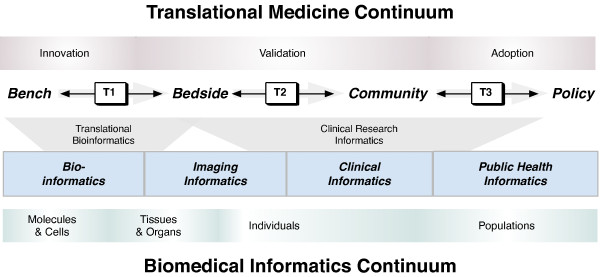
**The synergistic relationship across the biomedical informatics and translational medicine continua**. Major areas of translational medicine (along the top; innovation, validation, and adoption) are depicted relative to core focus areas of biomedical informatics (along the bottom; molecules and cells, tissues and organs, individuals, and populations). The crossing of translational barriers (T1, T2, and T3) can be enabled using translational bioinformatics and clinical research informatics approaches, which are comprised of methodologies from across the sub-disciplines of biomedical informatics (e.g., bioinformatics, imaging informatics, clinical informatics, and public health informatics).

The ultimate goal of translational medicine is the development of new treatments and insights towards the improvement of health across populations[11]. The first step in this process is the identification of *what *interventions might be worthy to consider[12]. Next, directed evaluations (e.g., randomized controlled trials) are used to identify the efficacy of the intervention and to provide further insights into *why *a proposed intervention works[12]. Finally, the ultimate success of an intervention is the identification of *how *it can be appropriately scaled and applied to an entire population[12]. The various contexts presented across the translational medicine spectrum enable a "grounding" of biomedical informatics approaches by providing specific scenarios where knowledge management and integration approaches are needed. Between each of these steps, *translational barriers *are comprised of the challenges associated with the translation of *innovations *developed through bench-based experiments to their clinical *validation *in bedside clinical trials, ultimately leading to their *adoption *by communities and potentially leading to the establishment of policies. The crossing of each translational barrier ("T1," "T2," and "T3," respectively corresponding to translational barriers at the bench-to-bedside, bedside-to-community, and community-to-policy interfaces; as shown in Figure 1) may be greatly enabled through the use of a combination of existing and emerging biomedical informatics approaches[9]. It is particularly important to emphasize that, while the major thrust is in the forward direction, accomplishments, and setbacks can be used to valuably inform both sides of each translational barrier (as depicted by the arrows in Figure 1). An important enabling step to cross the translational barriers is the development of trans-disciplinary teams that are able to integrate relevant findings towards the identification of potential breakthroughs in research and clinical intervention[13]. To this end, biomedical informatics professionals ("biomedical informaticians") may be an essential addition to a translational medicine team to enable effective translation of concepts between team members with heterogeneous areas of expertise.

Translational medicine teams will need to address many of the challenges that have been the focus of biomedical informatics since the inception of the field. What follows is a brief description of biomedical informatics, followed by a discussion of selected key topics that are of relevance for translational medicine: (1) Decision Support; (2) Natural Language Processing; (3) Standards; (4) Information Retrieval; and, (5) Electronic Health Records. For each topic, progress and activities in bio-, imaging, clinical and public health informatics are described. The article then concludes with a consideration of the role of biomedical informaticians in translational medicine teams.

## Biomedical Informatics

Biomedical informatics is an over-arching discipline that includes sub-disciplines such as bioinformatics, imaging informatics, clinical informatics, and public health informatics; the relationships between the sub-disciplines have been previously well characterized[7,14,15], and are still tenable in the context of translational medicine. Much of the identified synergy between biomedical informatics and translational medicine can be organized into two major categories that build upon the sub-disciplines of biomedical informatics (as shown in Figure 1): (1) translational bioinformatics (which primarily consists of biomedical informatics methodologies aimed at crossing the T1 translational barrier) and (2) clinical research informatics (which predominantly consists of biomedical informatics techniques from the T1 translational barrier across the T2 and T3 barriers). It is important to emphasize that the role of biomedical informatics in the context of translational medicine is not to necessarily create "new" informatics techniques[16]. Instead, it is to apply and advance the rich cadre of biomedical informatics approaches within the context of the fundamental goal of translational medicine: *facilitate the application of basic research discoveries towards the betterment of human health or treatment of disease*[17].

Clinical informatics has historically been described as a field that meets two related, but distinct needs[18]: patient-centric and knowledge-centric. This notion can be generalized for all of biomedical informatics within the context of translational medicine to suggest that the goals are either to meet the needs of user-centric stakeholders (e.g., biologists, clinicians, epidemiologists, and health services researchers) or knowledge-centric stakeholders (e.g., researchers or practitioners at the bench, bedside, community, and population level). *Bioinformatics *approaches are needed to identify molecular and cellular regions that can be targeted with specific clinical interventions or studied to provide better insights to the molecular and cellular basis of disease[19-25]. *Imaging informatics *techniques are needed for the development and analysis of visualization approaches for understanding pathogenesis and identification of putative treatments from the molecular, cellular, tissue or organ level[26-29]. *Clinical informatics *innovations are needed to improve patient care through the availability and integration of relevant information at the point of care[30-35]. Finally, *public health informatics *solutions are required to meet population based needs, whether focused on the tracking of emergent infectious diseases[36-39], the development of resources to relate complex clinical topics to the general population[40-44] or the assessment of how the latest clinical interventions are impacting the overall health of a given population[45-47].

At the T1 translational barrier crossing, *translational bioinformatics *is rapidly evolving with the enhancement and specialization of existing bioinformatics techniques and biological databases to enable identification of specific bench-based insights[16]. Similarly, *clinical research informatics*[48] emphasizes the use of biomedical informatics approaches to enable the assessment and moving of basic science innovations from the T1 translational barrier and across the T2 and T3 translational barriers (as depicted in Figure 1). These approaches may involve the enhancement and specialization of existing and new clinical and public health informatics techniques within the context of implementation and controlled assessment of novel interventions, development of practice guidelines, and outcomes assessment.

*Translational bioinformatics *and *clinical research informatics *are built on foundational knowledge-centric (i.e., "hypothesis-driven") approaches that are designed to meet the myriad of research and information needs of basic science, clinical, and public health researchers. The future of biomedical informatics depends on the ability to leverage common frameworks that enable the translation of research hypotheses into practical and proven treatments [49]. Progress has already been seen in the development of knowledge management infrastructures and standards to enable biomedical research to facilitate general research inquiry in specific domains (e.g., cancer[50] and neuroimaging[51]). It is also imperative for such advancements to be done in the context of improving user-centric needs, thereby improving patient care. To this end, the ability to manage and enable exploration of information associated with the biomedical research enterprise suggests that human medicine may be considered as the ultimate model organism [52]. Towards this aspiration, biomedical informaticians are uniquely equipped to facilitate the necessary communication and translation of concepts between members of trans-disciplinary translational medicine teams.

## Decision Support

Decision support systems are information management systems that facilitate the making of decisions by biomedical stakeholders through the intelligent filtering of possible decisions based on a given set of criteria [53]. A decision support system can be any computer application that facilitates a decision making process, involving at least the following core activities [54]: (1) *knowledge acquisition *- the gathering of relevant information from knowledge sources (e.g., research databases, textbooks, or experts); (2) *knowledge representation *- representing the gathered knowledge in a systematic and computable way (e.g., using structured syntax[55-57] or semantic structures[58,59]); (3) *inferencing *- analyzing the provided criteria towards the postulation of a set of decisions (e.g., using either rule based[60] or probabilistic approaches[61]); and, (4) *explanation *- describing the possible decisions and the decision making process.

The leveraging of computational techniques to aide in decision-making has been well established in the clinical arena for more than forty years[62]. In bioinformatics, a range of systems have been developed to support bench biologist decisions, including sequence similarity[63], *ab initio *gene discovery[64], and gene regulation[65]. There has been discussion of decision support systems that can incorporate genetic information in the providing of clinical decision support recommendations [66,67]. Decision support systems have been developed within imaging informatics for enabling better (both in terms of sensitivity and specificity) diagnoses of a range of diseases[68,69]. Clinical informatics research has given consideration to both positive and negative aspects of computer facilitated decision support [70-78]. Recent attention to bioterrorism planning and syndromic surveillance has also given rise to public health informatics solutions that involve significant decision support[79-81].

Decision support systems in the context of translational medicine will require a new paradigm of trans-disciplinary inferencing approaches to cross each of the translational barriers. Inherent in the design of such decision support systems that span multiple disciplines will be the need for collaboration and cross-communication between key stakeholders at the bench, bedside, community, and population levels. To this end, there may be utility in decision support systems incorporating "Web 2.0" technologies[82], which enable Web-mediated communication between experts across disciplines. Such technologies have begun to emerge in scenarios where expertise and beneficiaries are inherently distributed, such as rare genetic diseases[83]. Regardless of the approach chosen, the fundamental tasks of knowledge acquisition, representation, and inferencing and explanation will be required to be done with members of the translational medicine team. The successful design of translational medicine decision support systems could become an essential tool to bridge researchers and findings across biological, clinical, and public health data.

## Natural Language Processing

Natural Language Processing (NLP) systems fall into two general categories: (1) *natural language understanding *systems that extract information or knowledge from human language forms (either text or speech), often resulting in encoded and structured forms that can be incorporated into subsequent applications[84,85]; and, (2) *natural language generation *systems that generate human understandable language from machine representations (e.g., from within a knowledge bases or systems of logical rules)[86]. NLP has a strong relationship to the field of computational linguistics, which derives computational models for phenomena associated with natural language (encapsulated as either sets of handcrafted rules or statistically derived models)[87].

The development and application of NLP approaches has been a significant focus of research across the entire spectrum of biomedical informatics. Biological knowledge extraction has also been a major area of focus in NLP systems[88,89], including the use of NLP methods to facilitate the prediction of molecular pathways[90]. Within imaging informatics, there has been a range of applications that involve processing and generating information associated with clinical images that are often used to help summarize and organize radiology images[91-94]. In clinical informatics, there have been great advances in the extraction of information from semi-structured or unstructured narratives associated with patient care [95], as well as the development of applications for generating summaries or reports automatically[96-98]. In the realm of public health, NLP approaches have been demonstrated to facilitate the encoding and summarization of significant information at the population level, such as for describing functional status[99] and outbreak detection[100].

Peer-reviewed literature, such as indexed by MEDLINE, has been shown to be a source of previously unknown inferences across domains[101,102] as well as linkages between the bioinformatics and clinical informatics communities[103]. In addition to MEDLINE, which grows by approximately 1 million citations per year[104], the increasing adoption of Electronic Health Records will lead to increased volumes of natural language text[105]. To this end, NLP approaches will increasingly be needed to wade through and systematically extract and summarize the growing volumes of textual data that will be generated across the entire translational spectrum[106]. There has also been some work in NLP that directly strives to develop linkages across disparate text sources (e.g., bridging e-mail communications to relevant literature[107]). Within the realm of translational medicine, NLP approaches will be increasingly poised to facilitate the development of linkages between unstructured and structured knowledge sources across the realms of biology, medicine, and public health.

## Standards

The task of transmitting or linking data across multiple biomedical data sources is often difficult because of the multitude of different formats and systems that are available for storing data. Standard methods are thus needed for both representing and exchanging information across disparate data sources to link potentially related data across the spectrum of translational medicine [108]- from laboratory data at the bench to patient charts at the bedside to linkage and availability of clinical data across a community to the development of aggregate statistics of populations. These standards need to accommodate the range of heterogeneous data storage systems that may be required for clinical or research purposes, while enabling the data to be accessible for subsequent linkage and retrieval. Standards are thus an essential element in the *representation *of data in a form that can be readily *exchanged *with other systems.

The development of standards to represent and exchange data has been a major area of emphasis in biomedical informatics since the 1980's[108-113]. Much energy has been placed in the development of knowledge representation constructs[109,114,115] (e.g., ontologies and controlled vocabularies), as well as establishment of standards for their use and incorporation in biological[116], clinical[117,118], and public health[119] contexts. For example, the voluminous data associated with gene expression arrays gave rise to the Minimum Information About Microarray Experiment (MIAME) standard by the bioinformatics community[120]. Within the imaging informatics community, the Digital Imaging and COmmunications in Medicine (DICOM) defines the international standards for representing and exchanging data associated with medical images[121]. Within the clinical realm, Health Level 7 (HL7) standards are commonplace for describing messages associated with a wide range of health care activities[122,123]. Specific clinical terminologies, such as the Systematized Nomenclature of Medicine-Clinical Terms (SNOMED CT) can be used to represent, with appropriate considerations[124,125], clinical information associated with patient care. Data standards have been developed for systematically organizing and sharing data associated with clinical research[112,126], including those from HL7 and the Clinical Data Standards Interchange Consortium (CDISC). Within public health, the International Statistical Classification of Diseases and Related Health Problems (ICD) is a standard established by the World Health Organization (WHO) and used in the determination of morbidity and mortality statistics[127]. The rapid emergence of regional health information exchange networks has also necessitated that a range of standards be used to ensure the interoperability of clinical data[128-133]. The Comité Européen de Normalisation in collaboration with the International Organization for Standardization (ISO) is coordinating the common representation and exchange standards across the clinical and public health realms (through ISO/TC 215[134]).

The re-use of data in the development and testing of research hypotheses is a regular area of interest in biomedical informatics[126,135]. However, disparities between coding schemes pose potential barriers in the ability for systematic representation across biomedical resources[136]. Furthermore, the development of new representation structures is becoming increasingly easier[137], resulting in many possible contextual meanings for a given concept. The Unified Medical Language System (UMLS) [138] has demonstrated how it may be possible to develop conceptual linkages across terminologies that span the entire translational spectrum[139], from molecules to populations[114]. Additional centralized resources have been developed that facilitate the development and dissemination of knowledge representation structures that may not necessarily be part of the UMLS (e.g., the National Center for Biomedical Ontology[140] and its BioPortal[141]).

Standards that have been developed and are implemented by the biomedical informatics community will be an essential component towards the goal of integrating relevant data across the translational barriers (e.g., to answer questions like what is the comparative effectiveness of a particular pharmacogenetic treatment versus conventional pharmaceutical treatments in the general population?). Additionally, standards can facilitate the access and integration of information associated with a particular individual in light of available biological, imaging, clinical, and public health data (including improved access to these data from within medical records), ultimately enabling the development and testing the utility of "personalized medicine." Consequently, translational medicine will depend on biomedical informatics approaches to leverage existing standards (e.g., MIAME, HL7, and DICOM) and resources like the UMLS, in addition to developing new standards for specialized domains (e.g., cancer[142] and neuroimaging[143]).

## Information Retrieval

Information retrieval systems are designed for the organization and retrieval of relevant information from databases. The basic premise is that a query is presented to a system that then attempts to retrieve the most relevant items from within database(s) that satisfy the request[144]. The quality of the results is then measured using statistics such as precision (the number of relevant results retrieved relative to the total number of retrieved results) and recall (the number of relevant results retrieved relative to the total number of relevant items in the database).

Across the field of biomedical informatics, various efforts have focused on the need to bring together information across a range of data sources to enable information retrieval queries[145,146]. Perhaps the most popular information retrieval tool is the PubMed interface to the MEDLINE citation database that contains information across much of biomedicine[147]. In addition to MEDLINE, the growth of publicly available resources has been especially remarkable in bioinformatics[148], which generally focus on the retrieval of relevant biological data (e.g., molecular sequences from GenBank given a nucleotide or protein sequence). Information retrieval systems have also been developed in bioinformatics that are able to retrieve relevant data from across multiple resources simultaneously (e.g., for generating putative annotations for unknown gene sequences[149]). Imaging information retrieval systems have been a rich research area where relevant images are retrieved based on image similarity[150] (e.g., to identify pathological images that might be related to a particular anatomical shape and related clinical context[151]). Within clinical environments, information retrieval systems have been developed that can link users to relevant clinical reference resources based on using the particular clinical context as part of the query (e.g., to identify relevant articles based on a specific abnormal laboratory result)[152,153]. Information retrieval systems have been developed in public health to identify relevant information for consumers, epidemiologists, and health service researchers given varying types of queries[47,154,155]. The procedural tasks involved with information retrieval often involve natural language processing and knowledge representation techniques, such as highlighted previously. The integration of natural language processing, knowledge representation, and information retrieval systems has led to the development of "question-answer" systems that have the potential to provide more user-friendly interfaces to information retrieval systems[156].

The need to identify relevant information from multiple heterogeneous data sources is inherent in translational medicine, especially in light of the exponential growth of data from a range of data sources across the spectrum of translational medicine. Within the context of translational medicine, information retrieval systems could be built on existing and emerging approaches from within the biomedical informatics community, including those that make use of contemporary "Semantic Web" technologies[157-159]. The ability to reliably and efficiently identify relevant information, such as demonstrated by archetypal information retrieval systems developed by the biomedical informatics community (e.g., GenBank and MEDLINE), will be crucial to identify requisite knowledge that will be necessary to cross each of the translational barriers.

## Electronic Health Records

Medical charts contain the sum of information associated with an individual's encounters with the health care system. In addition to data recorded by direct care providers (e.g., physicians and nurses), medical charts typically include data from ancillary services such as radiology, laboratory, and pharmacy. With the increasing electronic availability of data across the health care enterprise, paper-based medical charts have evolved to become computerized as Electronic Health Records (EHRs). EHRs can capture a variety of information (e.g., by clinicians at the bedside) and have electronic interfaces to individual services (e.g., administrative, laboratory, radiology, and pharmacy). Many EHRs can enable Computerized Provider Order Entry (CPOE), which allows clinicians to electronically order services and may also enable real-time clinical decision support (e.g., provide an alert about an order that could lead to an adverse event[160]). Clinical documentation can be entered directly into EHR systems, allowing for potentially fewer issues due to transcription delays or difficulty in deciphering handwritten notes. An artifact of EHRs is the development of more robust clinical and research data warehouses, which can be used for subsequent studies[161-163].

From the earliest propositions of electronic health records[164,165], it has been thought that the potential benefits to support and improve patient care would been immense[166]. From a bioinformatics perspective, the integration of genomic information in EHRs may lead to genotype-to-phenotype correlation analyses[167,168], and thus increase the importance of bioinformatics integration with laboratory and clinical information systems[169]. The ability to review radiological images or search for possible clinically relevant features within them has shown great promise by the imaging informatics community[170-174]. Recent attention to EHRs has been given by the United States federal government as a core element of the modern reformation of health care[175]. Empirical studies will be needed to demonstrate the actual implications on patient care and effects on the reduction in overall health care costs as a direct result of EHR implementation[176,177]; however, there remains great interest in overall benefit of patient care and management to keep up with the dizzying pace of modern medicine within the clinical informatics community[176,178,179], including the development of integrated clinical decision support systems[66]. Public health informatics initiatives have pioneered surveillance projects for outbreak detection[180,181] or patient safety[182,183] that involve EHRs (which are also noted for their potentially high costs of implementation[184]). Recently, energy has also focused on the development of personal health records (PHRs) as a means to extend the realm of clinical care beyond the clinic into patient homes[185]. Through PHRs, consumers can be directly involved with their care management plans and as easily used as other electronic services (e.g., ATMs for banking[186] or using increasingly popular "Web 2.0" collaboration technologies[187]). Like EHRs, there is still need to assess the true benefits of PHRs in terms of their actual impact on the improvement of patient care[188,189]. The potential ubiquity of EHRs underscores the importance of considering the associated privacy and ethical issues (e.g., who has access to which kinds of data and for what purposes can clinical data actually be used for research or exchanged through regional interchanges)[189-193].

The increased availability of electronic health data, which are largely available and organized within EHRs, may have a significant impact on translational medicine. For example, the emergence of "personal health" projects (e.g., Google Health[117]) and consumer services (e.g., 23andMe[118]) has the potential to generate more genotype (i.e., "bench") and phenotype (i.e., "bedside") data that may be analyzed relative to community-based studies. The raw elements that could lead to the next breakthroughs may be made available as part of the data deluge associated with consumer-driven, "grass-roots" efforts. Such initiatives, in addition to the other core biomedical informatics topics discussed here (decision support, natural language processing, and information retrieval techniques), will enable the leveraging of EHR-based health data to catalyze the crossing of the translational barriers.

## The Role of the Biomedical Informatician in a Translational Medicine Team

Translational medicine is a trans-disciplinary endeavor that aims to accelerate the process of bringing innovations into practice through the linking of practitioners and researchers across the spectrum of biomedicine. As evidenced by major funding initiatives (e.g., the United States National Institutes of Health "Roadmap"[194,195]), there is great hope in the development of a new paradigm of research that catalyzes the process from bench to practice. The trans-disciplinary nature of the translational barrier crossings in translational medicine endeavors will increasingly necessitate biomedical informatics approaches to manage, organize, and integrate heterogeneous data to inform decisions from bench to bedside to community to policy.

The distinctions between multi-disciplinary, inter-disciplinary, and trans-disciplinary goals have been described as the difference between additive, interactive, and holistic approaches[196-198]. Unlike multi-disciplinary or inter-disciplinary endeavors, trans-disciplinary initiatives must be completely convergent towards the development of completely new research paradigms. The greatest challenge faced by translational medicine, therefore, is the difficulty in truly being a trans-disciplinary science that brings together researchers and practitioners that traditionally work within their own "silos" of practice.

**Figure 2:**
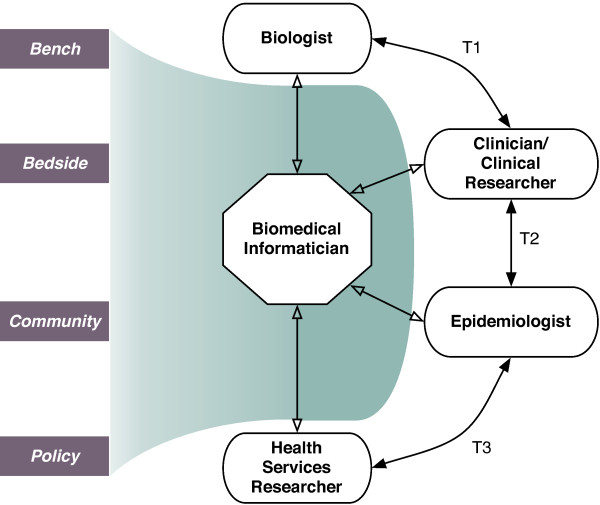
**The role of the biomedical informatician in a translational medicine team**. Biomedical informaticians interact with key stakeholders across the translational medicine spectrum (e.g., biologists, clinicians/clinical researchers, epidemiologists, and health services researchers). The suite of methods as described in this manuscript and depicted as the shadowed region enable the transformation of data from bench, bedside, community, and policy based data sources (shown in blocks).

Formally trained biomedical informaticians have a unique education[199-205], often with domain expertise in at least one area, which is specifically designed to enable trans-disciplinary team science, such as needed for the success within a translational medicine team. There is some discussion over what level of training constitutes the minimal requirements for biomedical informatics training[200,201,206-214], including discussion about what combination of technical and non-technical skills are needed[2,215]. However, a uniform feature of all formally trained biomedical informaticians is, as shown in Figure 2, their ability to interact with key stakeholders across the translational medicine spectrum (e.g., biologists, clinicians/clinical researchers, epidemiologists, and health services researchers). Furthermore, biomedical informaticians bring the methodological approaches (depicted as the shadowed region in Figure 2), such as the five topics highlighted in earlier sections of this article, which can enable the development and testing of new trans-disciplinary hypotheses. It is important to note that the topics discussed in this article are only a sampling of the full array of biomedical informatics techniques that are available (e.g., cognitive science approaches, systems design and engineering, and telehealth).

The success of translational medicine will depend not only on the addition of biomedical informaticians to translational medicine teams, but also on the acceptance and understanding of what biomedical informatics consists of by other members in the team. To this end, the importance of biomedical informatics training has been underscored as a key area of required competency across the spectrum of translational medicine, from biologists[216] to clinicians[217] to public health professionals[218]. There has been some demonstrable success in the development of experiences that focus on training "agents of change" with necessary core concepts[219] as well as hallmark distributed educational programs that aim to provide formal educational opportunities for biomedical informatics training[220]. The composition of translational medicine teams will also depend on the appropriate intermixing of biomedical informatics expertise to complement the requisite domain expertise[16]. To this end, the success of translational medicine endeavors may undoubtedly be greatly enhanced with biomedical informatics approaches; however, the appropriate synergistic relationship between biomedical informaticians and other members of the translational medicine team remains one of the next major challenges to be addressed in pursuit of translational medicine breakthroughs.

## Conclusion

Since its beginnings, biomedical informatics innovations have been developed to support the needs of various stakeholders including biologists, clinicians/clinical researchers, epidemiologists, and health services researchers. A range of biomedical informatics topics, such as those described in this paper, form a suite of elements that can transform data across the translational medicine spectrum. The inclusion of biomedical informaticians in the translational medicine team may thus help enable a trans-disciplinary paradigm shift towards the development of the next generation of groundbreaking therapies and interventions.

## Competing interests

The author declares that they have no competing interests.

## Authors' contributions

INS conceived of and drafted the manuscript as written.
